# Does Australia have a concussion ‘epidemic’?

**DOI:** 10.2217/cnc-2019-0015

**Published:** 2020-01-10

**Authors:** Elizabeth Thomas, Melinda Fitzgerald, Gill Cowen

**Affiliations:** 1 Program Lead, Centre for Clinical Research Excellence, Curtin University, Bentley, Australia; 2 Adjunct Research Fellow, Division of Surgery, Faculty of Health & Medical Sciences, The University of Western Australia, Perth, Australia; 3 Professor of Neurotrauma, Experimental & Regenerative Neurosciences, School of Biological Sciences, The University of Western Australia, Perth, Australia; 4 Professor of Neurotrauma, Curtin Health Innovation Research Institute, Curtin University, Bentley, Australia; 5 Professor of Neurotrauma, Perron Institute for Neurological & Translational Science, Sarich Neuroscience Research Institute Building, Nedlands, Australia; 6 Senior Lecturer, Curtin Medical School, Curtin University, Bentley, Australia

**Keywords:** concussion, economics, epidemiology, health

This editorial highlights the significant gaps in our understanding of concussion, identifying information necessary for further understanding of this clinical diagnosis. This would lead to improvement in management and allow appropriate resource allocation.

In the last decade, there has been significant attention paid by the media to the ‘silent epidemic’ of concussion [1]. This, in part, has been triggered by athletes sharing emotional perspectives [2] describing their experiences of the sustained effects of concussion. During this time, diagnostic assessment guidelines in sports have been regularly updated and there has been widespread dissemination of on- and off-field assessment tools and information regarding safe return-to-play [3,4]. An increase in concussion awareness in the sporting community [5] has followed. Sports-related concussion, however, represents only 16% of all annual concussive injuries in Australia [6], with motor vehicle accidents, falls and violent assaults representing the major contributors to concussion diagnoses.

## Epidemiology of concussion

There are significant gaps in our understanding of both sport-related and nonsport-related concussion. Gaps in our knowledge in the general population include incidence, prevalence, specific pathophysiology, reliable diagnosis, clear management pathways, prognosis and recovery patterns, and the direct and indirect economic burden on the health system. This lack of information results in an inability to confirm if concussion is indeed ‘an epidemic’ in Australia and prevents accurate assessment of the socioeconomic cost of concussion to society.

Although this information is necessary for both state and national level health resource allocation and service planning, clinicians are still left asking, ‘are we doing our best for concussed patients?’ and ‘can we be doing it more cost-effectively?’

In order to answer these questions, more accurate data on the current burden of disease is needed. The most current published national population data estimated 11,500 hospitalizations for concussion in Australia in 2004 and 2005 [7]. This does not account for concussion diagnoses made in the community, such as in general practice or similar primary healthcare facilities.

A proportion of cases of concussion will not present to a medical doctor, or will present but still go undiagnosed because the concussion was masked by a more serious co-occurring trauma. Hence, there is currently no Australian data establishing the true burden of concussion, thus limiting both research capacity and effective health service planning. Although this seems like fundamental data to collect, concussion is a clinical diagnosis and it suffers all the confounders and biases associated with a subjective variable.

## The cost of concussion

A 2013 analysis of routinely collected hospital admissions data in the state of Victoria reported the total direct cost of sports-related concussion in Victoria was around AU$2 million annually, giving a median cost per admission of AU$1583. Extrapolating to concussion from all causes nationally, and assuming the Victorian population represents close to a quarter of the Australian population, this equates to a direct health burden of around AU$50 million annually. This still does not account for patients presenting to a general practitioner, or indirect costs to the system such as allied health services and loss in productive life-years.

Furthermore, given that sports-related concussions make up a small percentage of concussive injuries, it is likely that the health burden is being grossly underestimated. Nonsport-related concussion is seen in typically complex populations such as children, the elderly, victims of assault and intimate partner violence and those in road traffic accidents. A greater set of comorbidities in such groups is also likely to increase costs.

Establishing the health and economic burden of concussion is further complicated by interinstitutional differences in medical record coding. The current ICD-10 code for concussion is S06.0, with five subcategorizations used to capture the duration of loss of consciousness (S06.00–S06.04). There is a risk that when translating from hospital discharge summaries, or in the case of multitrauma, miscoding or omission may occur. In general practice, coding is haphazard at best for non-notifiable conditions and while the diagnoses may exist in the body of text from a consultation, the data are largely irretrievable due to variation in documentation between clinicians.

## The lost sea of clinical data

In Australia, there are increasing numbers of private for-profit concussion clinics and there is increasing availability of online tools and apps aimed at increasing concussion awareness, diagnosis and directing patients to management resources. It has been reported overseas that significant money is spent by consumers paying for baseline testing and receiving postconcussion assessment and management advice through such companies [8]. To further increase our knowledge of the incidence and prevalence of concussion, there is a need for transparency surrounding data from such clinics in Australia and online services, including diagnostic methods and management techniques, and a need for such information to be coded in a standardized fashion. If the data from such private enterprises, along with data from general practice, private specialists and the public hospital system, could be captured via mandatory entry into a nationwide concussion registry then we may be able to start to answer the question ‘does Australia have a concussion “epidemic”?’

## The way forward

Concussion is a clinical diagnosis with variable presentations. To further understand this condition, improve diagnosis and management and establish its cost to society, long-term nationwide data collection is required, allowing for accurate identification of incidence and prevalence of concussion. A strategic approach to improve public hospital coding reliability, access to accurate coding from private hospital emergency departments and incentive for general practitioner’s to code using electronic medical records that make use of ICD-10 codes and have simple data extraction tools would be a valuable first step. De-identified data sharing from both public and private sectors should be the ultimate goal.
